# Factors associated with hospitalization times and length of stay in patients with bipolar disorder

**DOI:** 10.3389/fpsyt.2023.1140908

**Published:** 2023-05-19

**Authors:** Xiaoning Shi, Yingying Zhao, Haichen Yang, Xiufeng Xu, Yiru Fang, Xin Yu, Qingrong Tan, Huichun Li, Guangqiang Sun, Hang Wu, Pengfei Wang, Jie Yang, Xuequan Zhu, Gang Wang, Ling Zhang

**Affiliations:** ^1^Beijing Key Laboratory of Mental Disorders, National Clinical Research Center for Mental Disorders and National Center for Mental Disorders, Beijing Anding Hospital, Capital Medical University, Beijing, China; ^2^Advanced Innovation Center for Human Brain Protection, Capital Medical University, Beijing, China; ^3^Division of Mood Disorders, Shenzhen Mental Health Centre, Shenzhen, Guangdong Province, China; ^4^Department of Psychiatry, The First Affiliated Hospital of Kunming Medical University, Kunming, Yunnan, China; ^5^Division of Mood Disorders, Shanghai Mental Health Center, Shanghai Jiao Tong University School of Medicine, Shanghai, China; ^6^The Key Laboratory of Mental Health, National Clinical Research Center for Mental Disorders, Peking University Institute of Mental Health (The Sixth Hospital), Ministry of Health (Peking University), Beijing, China; ^7^Department of Psychiatry, Xijing Hospital, Fourth Military Medical University, Xi'an, Shanxi, China; ^8^The Second Affiliated Hospital, College of Medicine, Zhejiang University, Hangzhou, Zhejiang, China

**Keywords:** bipolar disorder, hospitalization, length of stay, risk factor, readmission

## Abstract

**Aim:**

Appraise the clinical features and influencing factors of the hospitalization times and length of stay in bipolar disorder (BD) patients.

**Methods:**

This is a multicenter, observational, cohort study of patients diagnosed of type I or type II bipolar disorder. Five hundred twenty outpatients in seven hospitals from six cities in China were recruited from February 2013 to June 2014 and followed up using a continuous sampling pattern. The research included a retrospective period of 12 months and the prospective period of 9 months. The demographic and clinical features of the patients were collected. The influencing factors that could affect the length of stay (number of days spent in the hospital in the prospective period) were analyzed by poisson's regression and the hospitalization times (times of hospitalization in the prospective and retrospective period) was analyzed by general linear model. The selected variables included gender, age, years of education, occupational status, residence status, family history of mental disease, comorbid substance abuse, comorbid anxiety disorder, times of suicide (total suicide times that occurred in the retrospective and prospective period), polarity of the first mood episode, and BD type(I/II).

**Results:**

Poisson's regression analysis showed that suicide times [Incidence Rate Ratio (IRR) = 1.20, *p* < 0.001], use of antipsychotic (IRR = 0.62, *p* = 0.011), and use of antidepressant (IRR = 0.56, *p* < 0.001) were correlated to more hospitalization times. Linear regression analysis showed that BD type II (β = 0.28, *p* = 0.005) and unemployment (β = 0.16, *p* = 0.039) which might mean longer duration of depression and poor function were correlated to longer length of stay. However, patients who experienced more suicide times (β = −0.21, *p* = 0.007) tended to have a shorter length of stay.

**Conclusion:**

Overall, better management of the depressive episode and functional rehabilitation may help to reduce the length of stay. BD patients with more hospitalization times were characterized by higher risk of suicide and complex polypharmacy. Patients at high risk of suicide tended to have inadequate therapy and poor compliance, which should be assessed and treated adequately during hospitalization.

**Clinical trial registration:**

www.ClinicalTrials.gov, Identifier: NCT01770704.

## 1. Introduction

Bipolar disorders (BD) are a group of chronic progressive mental disorders that include bipolar I disorder (BD I) and bipolar II disorder (BD II). The reported global lifetime prevalence rates are 0.6–1.0% for BD I and 0.4–1.1% for BD II (1). BD is associated with a worse quality of life, reduced psychosocial functioning, illness-related disability characterized by a high clinical overlap, and a chronic, relapsing longitudinal course (2).

The frequent recurrences of bipolar disorders are associated with poorer functioning, psychiatric and medical comorbidities, and increased odds of suicidality, disability, unemployment, and re-hospitalization. Increasing hospitalization rates is an unfavorable clinical outcome for BD, which would increase the utilization of healthcare services and bring a substantial financial burden for both medical services and patients (3).

Some studies investigated the predictors of recurrence in affective disorder. Several researchers have focused on the clinical outcomes in patients with BD and examined the factors associated with re-hospitalization and hospital length of stay. Hamilton et al. (3) found that housing instability, insurance coverage, and functional impairment could predict psychiatric re-hospitalization in BD patients. It is suggested that the length of stay is influenced by the patient's characteristics and external factors such as social characteristics. For example, lower functioning and longer duration of the current episode were predictors for the length of stay in adolescent inpatients with mood disorders (4). A retrospective study showed that functioning and pre-admission psychiatric polypharmacy predicted 30-day re-hospitalization in patients with BD-I (5). A naturalistic cohort study showed that remission at discharge was significantly associated with preventing re-hospitalization for inpatients with manic episode (6).

However, only a few studies have examined predictors of length of stay and hospitalization times in patients with BD (2, 4), and the key factors influencing length of stay and hospitalization times are still unclear. This was a multicenter cohort study of BD. More demographic and clinical features were included in this study. It was hypothesized that the characteristics that would act as predictors of hospitalization times and length of stay would include age, gender, years of education, occupational status, residence status, family history of mental disease, comorbid substance abuse, comorbid anxiety disorders, times of suicide, polarity of the first mood episode, and BD type(I/II). The objective of this study was to (1) evaluate the demographic and clinical characteristics that could affect the length of stay; (2) define the influencing factors of hospitalization times in the cohort of BD. Understanding the clinical and demographic risk factors for hospitalization in BD is helpful for close monitoring and could enhance the management of these patients.

## 2. Methods

### 2.1. Study participants and setting

This is a multicenter, observational, cohort study. The registration number is NCT01770704. The study was initiated by the Chinese Society of Psychiatry and was conducted in 7 major psychiatric hospitals in 6 cities of China between February 2013 and June 2014. Patients aged 18 years or older were recruited. All of the patients had a diagnosis of BD-I or BD-II according to DSM-IV criteria and had experienced at least one depressive or manic episode during the past 12 to 3 month before recruiting (7, 8). All patients signed informed consent including the retrospective cases. Patients who were unable to complete the patient questionnaire and/or who were participated in another interventional clinical study in the past year were excluded.

### 2.2. Assessment procedure

Patients diagnosed with BD who consented to participate in the study were referred by their clinical psychiatrists to the investigators. The study obtained approximately 21 months of follow-up data, including both the prospective and retrospective period. The retrospective period began 12 months before the study and ended when patients signed informed consent. The prospective study began when patients signed informed consent and ended 9 months after enrollment. Baseline visit was done when the patient signed the informed consent. The demographic information, medical history, and disease characteristics were recorded at baseline. In the prospective period, the treatment information, health resource consumption and clinical outcomes were collected at 1, 3, 6, and 9 months after the informed consent was signed. Data of hospitalization times, length of stay and demographic have been documented in the medical records, so no key data were missing. Data were recorded in the Case Report Form designed for the study.

The relative factors of selected variables that could affect the length of stay and hospitalization times were analyzed. Hospitalization times was defined as the total number of hospitalizations occurrences in the retrospective period (within the last 12 months) and the prospective period (within 9 months of follow-up). The Length of stay was defined as the days of hospitalization in the prospective period. The selected variables are as follows: gender, age, years of education, occupational status (employed/part-time/unemployed), residence status (living alone or together), family history of mental disorder, comorbid substance abuse, comorbid anxiety disorders, times of suicide, polarity of the first mood episode, and BD type (I/II).

In order to reduce the drop-out rates, the visits can be conducted by telephone and the time frame was set in the prospective visits. The study protocol was approved by the independent ethics committee of Beijing Anding Hospital and the respective hospitals.

### 2.3. Statistical analysis

The data were analyzed using SAS version 9.1. Hospitalization times and hospitalization length of stay each was expressed by mean ± standard and median [first quartile (Q1), third quartile (Q3)]. Poisson's regression analysis was used to determine the demographic and clinical variables associated with hospitalization times. Generalized linear models (GLM) analysis was employed to identify the variables related to the hospitalization length of stay. The level of significance was set at 0.05, two-tailed.

## 3. Results

### 3.1. Demographic and clinical characteristics of the sample

Five hundred fifty-five patients fulfilled the inclusion criteria and were invited to the study and 35 refused. Finally, 520 patients were included in the study. The demographic and clinical characteristics of the patients were presented in Table 1. Among the patients, 77% (*N* = 398) were diagnosed of bipolar I disorder. The average age of the sample was 35.65 ± 13.23 (mean ± SD). Most of them experienced depression (59%) and manic (33%) as polarity of the first mood episode (Table 1).

**Table 1:** Demographic and clinical characteristics of the sample.

**Variable**	**I & II (*N* = 520)**	**I (*N* = 398)**	**II (*N* = 122)**
**Gender**, ***N*** **(%)**			
Male	252 (48.46)	197 (49.50)	55 (45.08)
Female	268 (51.54)	201 (50.50)	67 (54.92)
Age, Mean ± SD	35.65 ± 13.23	35.23 ± 13.08	37.05 ± 13.67
Years of education, Mean ± SD	13.10 ± 3.40	13.20 ± 3.21	12.76 ± 3.96
**Occupational status**, ***N*** **(%)**			
Employed	221 (42.5)	166 (41.71)	55 (45.08)
Part-time	69 (13.27)	57 (14.32)	12 (9.84)
Unemployed	230 (44.23)	175 (43.97)	55 (45.08)
Living alone, *N* (%)	42 (8.08)	31 (7.79)	11 (9.02)
Comorbid substance abuse, *N* (%)	36 (6.92)	30 (7.54)	6 (4.92)
Times of suicide^*^, ***N*** **(%)**			
0	223 (42.88)	188 (47.24)	35 (28.69)
1	223 (42.88)	154 (38.69)	69 (56.56)
2	61 (11.73)	47 (11.81)	14 (11.48)
≥3	13 (2.51)	9 (2.26)	4 (3.27)
**Polarity of the first mood episode**, ***N*** **(%)**			
Manic	172 (33.08)	172 (43.21)	0 (0.00)
Hypomanic	19 (3.65)	7 (1.76)	12 (9.84)
Depression	307 (59.04)	197 (49.50)	110 (90.16)
Mixed	22 (4.23)	22 (4.23)	0 (0.00)

^*^Times of suicide with BD patients in the retrospective and prospective period. I, bipolar I disorder; II, bipolar II disorder.

Hospitalization times and length of stay with BD patients in the retrospective and prospective periods were shown in Table 2. Among all of the patients included in our study, 69% (*n* = 360) had been admitted to hospital among both respective and prospective periods, the mean time of hospitalization was 1.37 ± 0.64. In the prospective period 170 patients were admitted to hospital, the mean length of stay is 37 days with Q1 = 24 and Q3 = 56 (Table 2).

**Table 2:** Hospitalization times and length of stay with BD patients in the retrospective and prospective period.

**Period**	**I & II**	**I**	**II**
**R & P**			
*N*	360	289	71
T, Mean ± SD	1.37 ± 0.64	1.42 ± 0.68	1.20 ± 0.43
***P***			
*N*	170	133	37
T, Mean ± SD	1.09 ± 0.29	1.09 ± 0.29	1.11 ± 0.31
L, Median (Q1, Q3)	37 (24, 56)	36 (24, 53)	44 (25, 62)

T, Times of hospitalization in BD patients; P, Prospective period; R & P, Retrospective & Prospective period; L, Length of stay; I, bipolar I disorder; II, bipolar II disorder.

### 3.2. Factors associated with the times of hospitalization in patients with BD

Poisson's regression analysis showed that more times of suicide (times of suicide were divided to 0, 1, 2, ≥3, see Table 1) was associated with increasing risk of more hospitalization times (IRR = 1.20, *p* < 0.001). Psychopharmacological treatments also affected hospitalization times. Using of antipsychotics (IRR = 0.62, *p* = 0.010) and antidepressants (IRR = 0.56, *p* < 0.001) were influencing factors of more hospitalization times in the 12-month retrospective and 9-month follow-up period. No significant correlations were found in gender (IRR = 0.89, *p* = 0.163), age (IRR = 1.01, *p* = 0.984), living alone (IRR = 1.11, *p* = 0.507), comorbid of substance abuse (IRR = 0.99, *p* = 0.961) and comorbid of anxiety disorders (IRR = 1.08, *p* = 0.625). Polarity of the first mood episode (*p* > 0.05) and age at onset (IRR = 1.00, *p* = 0.709) also did not have significant correlation with times of hospitalization (Table 3).

**Table 3:** Factors associated with hospitalization times in BD patients.

**Variable**	**Estimate**	**IRR**	**CIL (95%)**	**CIU (95%)**	***P***
Age (≥65/ <65)	0.00	1.01	0.58	1.85	0.984
Gender (male/female)	1.95	0.89	0.76	1.05	0.163
Years of education	0.61	0.99	0.97	1.02	0.433
Living alone	0.44	1.11	0.82	1.56	0.507
Unemployed	0.74	1.08	0.91	1.28	0.389
Family history of mental disorder	0.34	0.95	0.79	1.13	0.562
Comorbid substance abuse	0.00	0.99	0.72	1.34	0.961
Comorbid anxiety disorders	0.24	1.08	0.79	1.43	0.625
BD type (I/II)	2.17	0.83	0.65	1.06	0.141
**Polarity of the first mood episode**					
Manic^*^	0.04	1.04	0.70	1.50	0.843
Hypomanic^*^	0.06	1.08	0.59	2.02	0.801
Depression^*^	0.23	1.10	0.74	1.58	0.633
Mixed^*^	—	—	—	—	—
Age at onset	0.14	1.00	0.98	1.01	0.709
Age at first diagnosed with BD	0.33	1.00	0.99	1.02	0.566
Suicide times	14.93	1.20	1.09	1.31	<0.001
With psychotic symptoms	3.71	1.18	1.00	1.41	0.054
Use of antipsychotics	6.52	0.63	0.43	0.88	0.011
Use of antidepressants	13.38	0.56	0.41	0.76	<0.001
Use of stabilizer	1.15	1.11	0.92	1.33	0.284

^*^Polarity of the first mood episode. IRR, Incidence Rate Ratio; CIL, Lower Confident Interval; CIU, Upper Confidence Interval.

### 3.3. Factors associated with the length of stay in patients with BD

Linear regression analysis showed that the longer length of stay was significantly associated with BD type II (β = 0.28, *p* = 0.005) and unemployment (β = 0.16, *p* = 0.039). However, patients who experienced more times of suicide (β = −0.21, *p* = 0.007) tended to have a shorter length of stay in the 9 months prospective period (Table 4). Polarity of any first mood episode did not affect the length of stay (*p* < 0.05). Gender (β = −0.10, *p* = 0.204), age (β = −0.14, *p* = 0.075), living alone (β = −0.05, *p* = 0.498), comorbid of substance abuse (β = −0.04, *p* = 0.585) and anxiety disorder (β = −0.03, *p* = 0.728) did not have correlation with the length of stay.

**Table 4:** Factors associated with length of stay in BD patients.

**Variable**	**b**	**β**	***p***
Age (≥65/ <65)	−290.21	−0.14	0.075
Gender (male/female)	−52.14	−0.10	0.204
Years of education	−10.41	−0.13	0.106
Living alone	−69.09	−0.05	0.498
Unemployed	89.18	0.16	0.039
Family history of mental disorder	−5.30	−0.01	0.911
Comorbid substance abuse	−41.39	−0.04	0.585
Comorbid anxiety disorders	−30.14	−0.03	0.728
BD type (I/II)	182.88	0.28	0.005
**Polarity of the first mood episode**			
Manic^*^	47.03	0.08	0.639
Hypomanic^*^	111.34	0.05	0.539
Depression^*^	17.97	0.03	0.855
Mixed^*^	—	—	—
Age at onset	7.35	0.34	0.057
Age at first diagnosed with BD	−2.45	−0.12	0.515
Hospitalization times	−6.75	−0.02	0.828
Suicide times	−82.42	−0.21	0.007
With psychotic symptoms	37.99	0.07	0.381
Use of antipsychotic	75.17	0.06	0.408
Use of antidepressant	94.48	0.17	0.064
Use of stabilizer	−24.16	−0.02	0.784

^*^Polarity of the first mood episode.

## 4. Discussion

Hospitalization is an essential aspect of the therapeutic strategy for BD, however, the patients with BD often require re-hospitalization, which can be a social-economic burden. To date, few studies have investigated the predictors of length of stay and hospitalization times in patients diagnosed of BD. The results of this study demonstrate that more suicide times, antipsychotic use, and antidepressant use are correlated with more times of hospitalization times, which means higher risk of suicide and more complex treatment. Patients diagnosed of BD type II or unemployed were found to have longer length of stay. On the other side, patients who experienced more times of suicide tended to have a shorter length of stay, which indicated inadequate therapy and poor compliance.

Benarous et al. (4) found that longer duration of current episode and worse functioning at admission were independent predictors for length of stay in adolescent patient of mood disorder. In our research, patients with occupational status of unemployed tended to have longer length of stay. Occupational status may have a relationship with cognitive and psychosocial functioning. Full-time employment is a symbol of high psychosocial functioning (9). Psychosocial functioning reflects a person's ability to perform daily life activities and establish relationships. Previous research found that neurocognitive deficits are predictors of the long-term clinical course among BD patients (10). Unemployment may be associated with poor psychosocial outcomes and need more time for recovery that resulted in a longer length of stay.

BD type II diagnosis also predicts a longer length of stay. Serra et al. (11) identified that BD-II diagnosis predicted long-term morbidity in BD and the total months of illness during follow-up were longer in BD-II than in BD-I subjects. Duration time of depression almost reached two-fold longer in BD-II than in BD-I; BD-II subjects spent 42% more time ill overall than BD I (11). The number of depressive episodes in the past year is a potential predictor of worse treatment outcomes, which may cause a longer length of stay (12).

We found that use of antipsychotics and antidepressants is associated with more hospitalizations. The previous study showed a positive association between hospitalization length of stay and the current treatment of antidepressants and second-generation antipsychotics (SGAs) among re-admitted treatment-resistant BD inpatients (13). Complex polypharmacy is very prevalent in the treatment of BD. However, previous research found that BD patients with complex polypharmacy had poor adherence to therapy and were less likely to respond clinically (14). Sussman et al. (15) showed that compared to BD I patients without antidepressant use, those with antidepressant use had 1.43 to 1.50 times the odds of re-hospitalization in the follow-up period. Among adolescence patients with BD, it was also found that using of SGAs and SSRIs was positively associated with hospitalization (16). Therefore, clinicians should increase medication adherence prior to adding another agent to medication regimens.

BD patients have a higher risk of suicide and are 20–30 times more likely to die by suicide than the general population (17). It was reported that about 0.9% of BD patients attempt suicide each year (18). In a 12-month prospective study, the researchers found that patients of BD type I with relapses showed more frequent suicide acts (2). However, patients with a high risk of suicide may have poor adherence to treatment. Our result showed that patients with more suicides have more hospitalization times but a shorter length of stay which might mean inadequate treatment during hospitalization. As a result, enhanced management should be taken for inpatients with high risk of suicide to improve the clinical treatment effect. Adequate clinical assessments and short-term interventions are needed, including electroconvulsive therapy (19), close clinical supervision, and a longer length of stay to lower the risk of suicide and re-hospitalization. This feature may indicate that BD patients with higher suicide risk require more comprehensive discharge planning and support.

### 4.1. Clinical implication and public policy recommendation

Re-hospitalization due to a relapse of BD is a financial burden for both medical services. Understanding the factors associated with the hospitalization helps to take measures that could reduce the hospitalization rate and the occupation of medical resources. Our results found that more suicide times and use of antipsychotics and antidepressants were correlated with more hospitalization times, which means that attention need be paid to patients with high risk of suicide and complex polypharmacy. On the other hand, BD type II, unemployed and more suicide times were correlated with the longer length of stay. In this situation, patients may suffer of more depressive episodes, impaired social functioning which might predict worse treatment outcomes and lead to longer length of stay. Improved management of the depressive process and functional rehabilitation may help to reduce the length of stay. Patients with high risk of suicide tend to have more hospitalization times but a shorter length of stay, which might indicate inadequate therapy and poor compliance. These patients should be assessed and treated adequately during hospitalization.

### 4.2. Limitation

The main strength of this study is that it was a multicenter cohort study conducted in a real world setting with the aim of clarifying the relationships between multiple predictors and hospitalization times and length of stay. However, there are also several limitations to this study. Certain limitations of our study should be considered. First, a larger sample size and a longer follow-up period could provide more reliable results; Second, the impacts of comorbidity with physical diseases, functioning, the severity of the patient's condition and treatment compliance were not evaluated. Third, as potential predictors of hospitalization, the effects of combining with non-drug therapies were not measured, such as modified electroconvulsive therapy (MECT), repetitive transcranial magnetic stimulation (r-TMS), and psychotherapy. Last, the data collected from 2013 to 2014 seem old. However, this research was a multicenter study and the sample size was relatively large. Therefore, it might be representative of the overall mental health situation at a certain time. The conclusions of this study were beneficial for the management of BD patients and could probably provide effective measures to reduce the utilization of hospitalization resources.

### 4.3. Conclusion

This study tried to determine the factors related to using mental health resources in BD patients. In conclusion, our study showed that suicide times and use of antipsychotics and antidepressants were significantly associated with hospitalization times, whereas BD type II, unemployed and more suicide times were significantly associated with longer length of stay. Other variables, including gender, age, years of education, living alone, age at onset, family history of mental disorder, comorbid substance abuse, comorbid of anxiety disorder, polarity of the first mood episode did not significantly affect the hospitalization times and length of stay. Further studies are needed to validate our results.

## Data availability statement

The original contributions presented in the study are included in the article/supplementary material, further inquiries can be directed to the corresponding author.

## Ethics statement

The studies involving human participants were reviewed and approved by Research Ethics Committee of Beijing Anding Hospital, Capital Medical University, Beijing, China. The patients/participants provided their written informed consent to participate in this study.

## Author contributions

Study design: GW and XY. Data collection, analysis, and interpretation: LZ, HY, XX, YF, QT, and HL. Drafting of the manuscript: XS, YZ, GS, and HW. Critical revision of the manuscript: LZ. All authors approved the final version for publication.
